# Effect of feed restriction on the maintenance energy requirement of broiler breeders

**DOI:** 10.5713/ab.21.0183

**Published:** 2021-10-29

**Authors:** Guilherme Ferreira da Silva Teofilo, Rony Riveros Lizana, Rosiane de Souza Camargos, Bruno Balbino Leme, Freddy Alexander Horna Morillo, Raully Lucas Silva, João Batista Kochenborger Fernandes, Nilva Kazue Sakomura

**Affiliations:** 1 Department of Animal Science, Faculty of Agricultural and Veterinarian Sciences of São Paulo State University – UNESP, Jaboticabal-SP, 14884-900, Brazil

**Keywords:** Basal Metabolic Rate, Fasting Heat Production, Feed Regimen

## Abstract

**Objective:**

This study aimed to evaluate the effect of the *ad libitum* and restricted feeding regimen on fasting heat production (FHP) and body composition.

**Methods:**

Twelve Hubbard broilers breeders were selected with the same body weight and submitted in two feeding regimes: Restricted (T1) with feed intake of 150 g/bird/d and *ad libitum* (T2). The birds were randomly distributed on the treatments in two runs with three replications per treatment (per run). The birds were adapted to the feed regimens for ten days. After that, they were allocated in the open-circuit chambers and kept for three days for adaptation. On the last day, oxygen consumption (VO_2_) and carbon dioxide production (VCO_2_) were measured by 30 h under fasting. The respiratory quotient (RQ) was calculated as the VCO_2_/VO_2_ ratio, and the heat production (HP) was obtained using the Brower equation (1985). The FHP was estimated throughout the plateau of HP 12 hours after the feed deprivation. The body composition was analyzed by dual-energy X-ray absorptiometry scanning at the end of each period. Data were analyzed for one-way analysis of variance using the Minitab software.

**Results:**

The daily feed intake was 30 g higher to T2 (p<0.01) than the T1. Also, the birds of the T2 had significatively (p<0.05) more oxygen consumption (+3.1 L/kg^0.75^/d) and CO_2_ production (+2.2 L/kg^0.75^/d). That resulted in a higher FHP 359±14 kJ/kg^0.75^/d for T2 than T1 296±17.23 kJ/kg^0.75^/d. In contrast, the RQ was not different between treatments, with an average of 0.77 for the fasting condition. In addition, protein and fat composition were not affected by the treatment, while a tendency (p<0.1) was shown to higher bone mineral content on the T1.

**Conclusion:**

The birds under *ad libitum* feeding had a higher maintenance energy requirement but their body composition was not affected compared to restricted feeding.

## INTRODUCTION

The genetic selection to the fast growth of broilers resulted in a proportional increase of feed intake by broiler breeders, causing a greater intake of nutrients and energy above the energy requirements for production and maintenance [1]. The high feed intake increases the fat deposition, carrying to body weight (BW) increase [2–4]. Due to higher feed intake, several metabolic disturbances can occur and reduce egg production, fertility, and hatchability [2–5]. According to Renema and Robinson [6], to reduce the excessive weight gain and incidence of reproductive disorders, broiler breeders must be submitted to restricted feeding programs. This feeding system aims to meet birds' nutritional requirements by providing reduced feed to control the weight variation and favour sexual development [2,7]. For this, the calculation of dietary nutrients must consider the amount of feed allocated, obtaining an adequate balance of nutrients and energy [8]. Also, adequate body composition should be achieved so the birds can withstand high reproductive rates [9]. In this way, limiting the regulation of feed intake according to their nutritional needs becomes critical. It depends exclusively on the feeding strategies adopted by specialists.

On the other hand, *ad libitum* feeding can be an alternative, where the diets' energy and nutritional density must be adjusted (qualitative restriction) to prevent over-feeding. Still, this feeding system presents several disadvantages, once the weight and body composition variations lead to alteration in the dynamic of energy metabolism and, consequently, in maintenance utilization [10,11]. Therefore, it was hypothesized that the feed regimen, depending on the previous feed intake level, affects the maintenance requirement measured throughout fasting heat production (FHP).

The determination of energy requirement for maintenance through the FHP [12] was barely described in poultry, and principally it was estimated with *ad libitum* fed birds. Thus, there is little information available to broiler breeders. Based on this, the broad understanding of bird's metabolic variations on feed restriction will favour the correct determination of maintenance, supporting future studies in the dynamic of energy metabolism studies in broiler breeders.

We aimed to evaluate the effect of feed regimen (restricted and *ad libitum*) on the FHP in broiler breeders in the production phase and describe their body components variation (protein, lipids, and minerals).

## MATERIALS AND METHODS

### Facilities and animal management

The study was conducted at the Poultry Science Laboratory (LAVINESP) at São Paulo State University, Campus Jaboticabal-SP. The animal utilization, management, and procedures were approved by The Ethics Committee on Animal Use (CEUA) of the Faculdade de Ciências Agrárias e Veterinárias, UNESP, Jaboticabal, São Paulo, Brazil under protocol no. 013079/19.

The assay was carried out using thirty Hubbard broiler breeders of 30 week-old. During the pre-experimental period, birds were individually allocated in cages to evaluate their productive performance. Twelve birds were selected at the peak of production from this group according to the BW and laying rate. The environmental temperature was adjusted to maintain around 22°C±2°C to guarantee thermal comfort, and the light program was adopted to provide 16 L:8 D.

During the pre-experimental period, the diet mash-type was daily provided in the morning (8:00 h) in a controlled manner (150 g/bird), and in the afternoon (16:00 h) the egg production was recorded. In addition, the birds were individually weighed each week. The basal diet was formulated to meet or exceed the nutritional requirements of birds (12.21 metabolizable energy MJ/kg and 15.5 crude protein).

### Experimental design and procedure

The selected birds were randomly distributed in two treatments according to feed regimen. T1, Restricted feeding (150 g/bird/d); and T2, *ad libitum* feeding (feed allocation of 300 g/bird/d, amount of feed sufficient to guarantee that the feeder would not be empty until the next feed supply).

**Figure 1:**
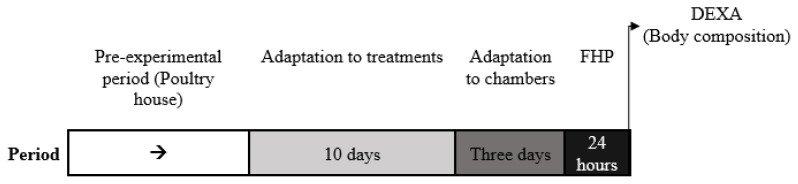
Protocol of experimental period for adaptation (treatments and chambers), and measurements. FHP, fasting heat production; DEXA, dual-energy X-ray absorptiometry.

Six birds per run were allocated individually in respirometry chambers for gas exchange measurements (oxygen consumption, VO_2_; and carbon dioxide production, VCO_2_). The indirect calorimetry system in our laboratory consists of six chambers on an intermittent reading throughout the day. For that reason, the measurement was divided into two runs with an interval of two weeks between runs. Hence the data collection was executed in two different runs with three replications per treatment in each run according to the following protocol (Figure 1): adaptation period to the chambers (3 d), measurement of heat production after the feed was withdrawal in fasting condition (24 h).

### Indirect calorimetry and heat production calculation

**Figure 2:**
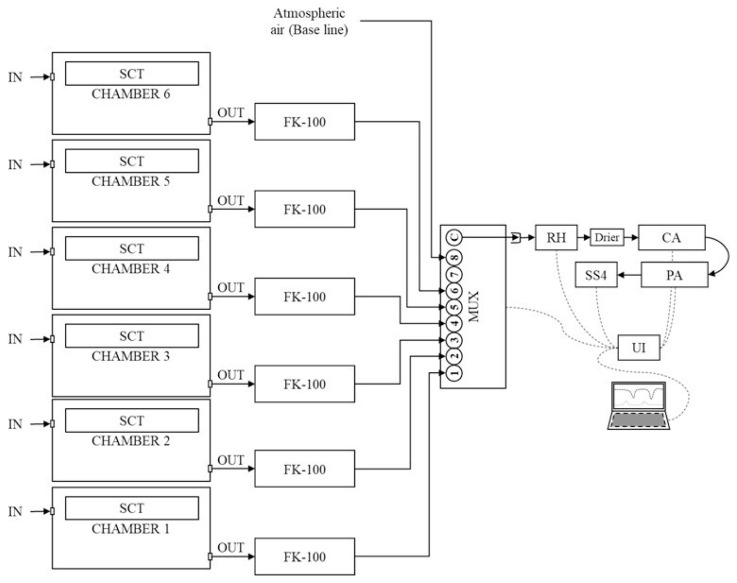
Components and operation diagram of the LAVINESP indirect calorimetry system. IN, air ingoing; SCT, temperature control system; OUT, air outgoing; FK-100, flow kit pump; MUX, Multiplexer (signal alternator); RH, Water vapor pressure analyzer; Drier, sample air drier; CA, carbon dioxide (CO_2_) analyzer; PA, paramagnetic oxygen analyzer (O_2_); SS4, sub-sampled air; UI, universal interface. Air flow direction (→) and data transference line (‐‐‐).

The indirect calorimetry facilities consist of an open-circuit system, with six chambers (dimension: 90×85×95 cm) equipped with an environmental temperature controller and a Sable System Classic Line (Sable System, North Las Vegas, NV, USA) pumps and gas analyzer detailed in Figure 2. The gas concentrations were analyzed using a carbon dioxide analyzer (CO_2_, %), following the principle of non-dispersed infrared absorbance and oxygen (O_2_, %) using a paramagnetic sensor. The gas exchange measurement was recorded each second and averaged by minute for heat production calculations.

The gas exchange calculation was according to the Lighton [13] description for an open-circuit system. The in-going airflow (F_in_) to the chamber, oxygen consumption (VO_2_), and production of carbon dioxide (VCO_2_) were computed according to the following calculations.


$$\begin{array}{l} {\text{F}}_{\text{in}}\;(\text{L}/\text{min})={\text{F}}_{\text{out}}\times (100-{\left[{\text{O}}_{2}\right]}_{\text{out}}-{\left[{\text{CO}}_{2}\right]}_{\text{out}})/(100-{\left[{\text{O}}_{2}\right]}_{\text{in}}-{\left[{\text{CO}}_{2}\right]}_{\text{in}})\\ {\text{VO}}_{2}\;(\text{L}/\text{min})={\text{F}}_{\text{in}}\times {\left[{\text{O}}_{2}\right]}_{\text{in}}-{\text{F}}_{\text{out}}\times {\left[{\text{O}}_{2}\right]}_{\text{out}}\\ {\text{VCO}}_{2}\;(\text{L}/\text{min})={\text{F}}_{\text{out}}\times {\left[{\text{O}}_{2}\right]}_{\text{out}}-{\text{F}}_{\text{in}}\times {\left[{\text{CO}}_{2}\right]}_{\text{in}} \end{array}$$

Where F_out_ is the outgoing airflow, [O_2_]_in_ and [CO_2_]_in_ are the atmospheric gas concentrations (baseline). VO_2_ and VCO_2_ were calculated from the corrected volume of gas at standard temperature and pressure dry and expressed by a unit of metabolic BW per day (kg^0.75^/d). The respiratory quotient (RQ) was calculated from VCO_2_/VO_2_, and the heat production (HP) was obtained by the Brouwer [14] equations:


$$\text{HP }(\text{kJ}/{\text{kg}}^{0.75}/\text{d})=16.18\times {\text{VO}}_{2}+5.02\times {\text{VCO}}_{2}$$

### Body composition analyses

After FHP measurements, lean mass (protein, water, and solute contents), lipid mass, and bone mineral content were analyzed for each bird *in vivo* using dual-energy X-ray absorptiometry (DEXA). The DEXA scanning procedure was done according to the validated procedure reported by Gonçalves et al [15]. The relative weight was expressed in grams of component per kilo of BW.

### Statistical analysis

After the adaptation period, the BW was recorded at the beginning and finalized the fasting period. Then, the daily feed intake (before the fasting period), gas exchange parameters, heat production, and body composition were analyzed through one-way analysis of variance, considering a 95% confidence interval. The lineal additive model used for the response data were as follow:


$${\text{Y}}_{\text{ij}}=\mu +\tau_{\text{i}}+{ɛ}_{\text{ij}}$$

Where: Y_ij_ is the observed variable, μ is the general mean, τ_i_ is the treatment effect, and ɛ_ij_ is the experimental error.

The FHP, RQ, VO_2_, and VCO_2_ of each bird (individually observed) and runs were calculated on the plateau phases of the HP behaviour during the fasting period. It was described around the last 8 hours of the fasting period (from 16 to 24 h of the fasting).

A segmented model was fit to the HP during the fasting period, considering the individual observation of each treatment according to the following model:


HP=U×(t<R)×(R-t)+L

Where U is the decline rate of HP until time R (broken point), L is the plateau value of HP (plateau-FHP), and t is the time. It was made through the non-linear regression procedure aimed to reduce the error of fit.

All statistical analyses were performed using Minitab v.20 statistical software (Minitab Inc., StateCollege, PA, USA).

## RESULTS

The performance parameters are shown in Table 1. At the beginning of the adaptation period to the treatments, the initial BW was similar between the birds distributed for each treatment (p>0.05), with an average weight of 4.003±0.230 kg for all birds. Also, the BW after the fasting period showed a tendency (p<0.10) to have a higher weight of birds fed *ad libitum* (4.153±0.349 kg) than birds under restricted feed (3.839±0.160). On the other hand, weight variation between the beginning and the final fasting period was significantly different (p<0.01) between treatments. The *ad libitum* fed birds lost 53 g more than restricted treatment before 24 h of fasting. Additionally, the birds under *ad libitum* treatment significantly (p<0.01) increased daily feed intake by 17%. Because *ad libitum* groups have free access to feed and water, a higher variation in feed intake on *ad libitum* treatments (SD = ±13.12 g) was observed compared to restricted groups (148±2.97). Also, the feed intake under restricted regimen treatment was close to the amount of feed allocated.

The VO_2_, VCO_2_, and FHP were different (p<0.01) between treatments. *Ad libitum* birds have higher VO_2_ (+3 VO_2_/kg^0.75^/d) and VCO_2_ (+2 VCO_2_/kg^0.75^/d) consumption, and consequently higher FHP values (+63 kJ/kg^0.75^/d). However, the RQ was not different between treatments (p = 0.490) (Table 2).

The body composition is presented in Table 3. There was no statistical difference in lipid and lean mass (p>0.05). However, bone mineral content was slightly different (p = 0.045), with +2 g/kg of bone mineral content on the birds submitted to restricted compared to *ad libitum* feeding regimen.

**Figure 3:**
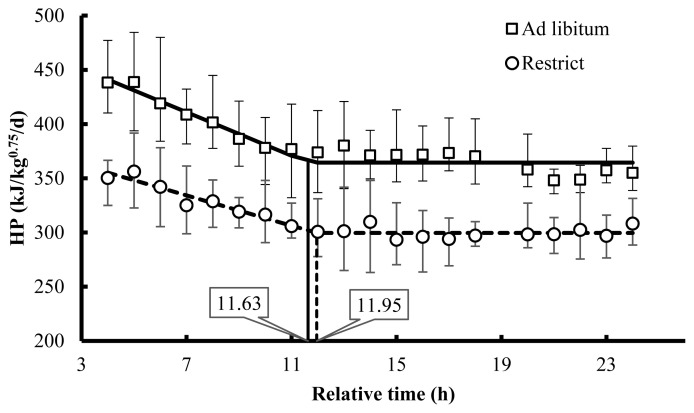
Broken line model [HP = U×(t<R)×(R−t)+L] of HP (heat production, kJ/kg^0.75^/d) along of 24 hours of fasting period of broiler breeders previously submitted to feed regimen of *ad libitum* (—) and restricted (‐‐‐).

The broken line model was fit for each treatment, and the parameters are presented in Table 4. Also, the HP (kJ/kg^0.75^/d) as a function of the time is illustrated in Figure 3. It was shown that the rate of energy expenditure declining after starting the fasting was higher on the *ad libitum* feeding, while the time to meet the broken point was the same, around 11 h 47 min in both treatments. As a result, the plateau of FHP was 64 kJ/kg^0.75^/d higher when the birds were fed *ad libitum*.

## DISCUSSION

The thirteen days that the birds were submitted to *ad libitum* feeding resulted in a BW gain of 314 g per bird, representing 7.5% more from the initial BW. An important characteristic to be considered in broiler breeders, compared to other poultry categories, is that the birds present a growth even when they are adults and in production [2,3]. Thus, these birds are characterized as having multiphasic growth [16], explaining why they had different growth rates during the adaptation period on both feeding regimes due to the different amounts of nutrient intake. While the feed restriction induced a slight BW loss, as Caldas et al [17] observed between 33 to 37 weeks of age, the group fed *ad libitum* increased BW by 132 g. Therefore, no control on feed intake and the BW can be carried to reproductive and sexual disorders affecting birds performance [1]. However, this depends on the length of the feed restriction period as hens can use body sources to recover nutrient needs for a certain period depending on the restriction level [2]. Also, under *ad libitum* feeding, with nutrient and energy intake above the recommendation [18], the individual feed intake variation leads to high variability in BW, making it difficult to get the flock's uniformity weight [19].

Gas exchange parameters have been scarcely described for broiler breeders during the production phase. For example, Caldas et al [17] reported recent results of 16.9 to 23 VO_2_ and 15.4 to 21.9 L/kg^0.75^/d for VCO_2_ measured in birds fed restricted programs. Also, the heat production ranged from 351 to 480 kJ/kg^0.75^/d, higher than we observed since the authors measures were conducted in fed birds, contrasted by the high RQ of 0.9 to 0.96. On the other hand, Anderson and Kulp [20], who made measurements 18 to 20 h after the feed was withdrawn, reported similar values to our study, VO_2_ (18.7 L/kg^0.75^/d), VCO_2_ (14.7 L/kg^0.75^/d), and RQ (0.82); which was close to the FHP (377 kJ/kg^0.75^/d) of birds on *ad libitum* treatment.

Besides, Misson et al [21] reported the same values of heat production (318 kJ/kg^0.75^/d), VO_2_ (16.1 L/kg^0.75^/d), and VCO_2_ (11.5 L/kg^0.75^/d) as those observed in the present study in birds on feeding restriction. However, the RQ was different (0.72) for a 24 h period of fasting. This could be due to the another strain (Light Sussex) and productive level used by the mentioned authors, which was different from the present study [22]. According to Salas et al [23] and Attia et al [2], broiler breeders during the peak of production have a high protein catabolism rate to prioritize the egg formation. Even under fasting conditions, this period was not enough to interrupt the egg production; the birds mobilize body reserve to supply nutrients, mainly energy, to form the next egg. Thus, this explains why the oxidation of proteins occurs, which is used to synthesize the egg and induce a higher RQ value [24].

A feed restriction induced a basal metabolic rate reduction in growing swine due to the body mass reduction [25], in growing chicks [10] and, calves [26]. It was explained by Spratt et al [27], who mentioned that the FHP variation due to the visceral mass is only 4%, and the significant fraction can be attributed to the body composition variation of around 30%. However, the lean and fat mass was not affected by the feed regimen treatments. These results were similar to those reported by Caldas et al [17], where birds of 30 weeks old were described with tissue contents of 186 g/kg for lean and 818 g/kg for fat mass. While the bone mineral content reported was 14 g/kg lower than those of our study. It is important to note that the methodology for determining body composition by DEXA has some limitations; lean mass is a conjunction between the protein and the water content that includes some mineral solutes. Therefore, it prevents a precise description of the actual value of body protein content since the water fraction is the factor that has more significant variability [28]. Additionally, DEXA is widely described by precision for determining mineral content. However, if there is a presence of an egg inside the animal, the mineral content would be influenced.

After feed deprivation in both groups, the metabolic rate behaviour was similar to reported by Zubair and Leeson [10] in 11 days-old broiler chicks, expressing a decline phases up to reach a minimum value described as plateau-FHP. Meanwhile, the broken point followed by the stable phases was reached at around 12 h of fasting in broiler breeders was not shown in growing birds. This metabolic phenomenon depends on the feed passage rate and gastrointestinal tract retention capacity, nutrient catabolism related to the body storage available, and demand for other metabolic functions. A significant reduction in HP is observed two hours after food deprivation due to the digestive tract is emptying, absorption of remaining nutrients, and metabolic adaptation to fasting conditions [26].

It was more evident in the *ad libitum* treatment, where birds presented a higher reduction of the heat production until the broken point. The time to reach the minimum stable phases of the heat production (or plateau-FHP) was the same between treatments. It is related to the feed intake amount and the bulk capacity of the broiler breeders. The broiler breeders bulk capacity is associated with the physic-chemical composition of feed and water retention that affects the passage rate [29]. As the same feed was offered, the bulk capacity was similar between treatments. The plateau value of HP along the fasting time has shown a difference of 64 kJ/kg^0.75^/d lower on the restricted birds group when compared with birds fed *ad libitum*. Similar results were observed by Zubair and Leeson [10], who reported a variation of 67 kJ/kg^0.75^/d on fasting. It corresponds to the energy expenditure of the body mass metabolic activity [30].

## CONCLUSION

Broiler breeders fed *ad libitum* had a high cost of basal energy expenditure and maintenance energy requirement in the production peak, without a variation in the body composition compared to those on restricted feeding.

## Figures and Tables

**Table 1:** Body weight and feed intake of broiler breeders with 30 weeks of age during the experimental period submitted to different feed regimens

Treatment	Initial BW (g/bird)^1)^	BW fasting (g/bird)^2)^	BW variation (g/bird)^3)^	FI (g/bird)^4)^
*Ad libitum*	4,021±257	4,153±349	216±19.73	173±13.12
Restricted	3,985±225	3,839±160	163±15.41	148±2.97
P	0.803	0.073	<0.01	0.001
RMSE	241	271	17.706	9.515

The result was presented as μ±standard deviation.

BW, body weight; FI, daily feed intake; P, probability; RMSE, root means square of the error.

1) Body weight at the start of the treatment adaptation period.

2) Body weight at the star the fasting period.

3) Difference between the initial and final body weight during the fasting period.

**Table 2:** Gas exchange parameters of oxygen consumption, CO_2_ production, respiratory quotient, and fasting heat production of broiler breeders submitted to the different feed regimens

Treatment	FHP (kJ/kg^0.75^/d)	RQ (VCO_2_/VO_2_)	VO_2_ (L/kg^0.75^/d)^1)^	VCO_2_ (L/kg^0.75^/d)^2)^
*Ad libitum*	359±14.71	0.779±0.033	18±0.77	14±0.668
Restricted	296±17.23	0.804±0.079	15±0.809	12±1.317
P	<0.010	0.49	<0.010	0.006
RMSE	16.022	0.061	0.789	1.045

The result was presented as μ±standard deviation. These values correspond to the last 8 hours of the fasting period (plateau of the heat production from 16 to 24 hours during the fasting period).

FHP, fasting heat production; RQ, respiratory quotient; P, probability; RMSE, root means square of the error.

1) Oxygen consumption.

2) CO_2_ production.

**Table 3:** Body composition (in g/kg) of broiler breeders submitted to different feed regimens

Treatment	BW (g/bird)^1)^	Fat mass	Lean mass	BMC (g/kg)
*Ad libitum*	3,933±350	194±28.6	806±28.6	20±1.866
Restricted	3,617±205	179±17.5	818±19.38	22±1.485
P	0.085	0.278	0.408	0.045
RMSE	287	23.675	24.405	1.687

The result was presented as μ±standard deviation.

BW, body weight; BMC, bone mineral content; P, probability; RMSE, root means square of the error.

1) Body weight value after fasting period.

**Table 4:** Parameters of the broken line model describe the metabolic rate variation (kJ/kg^0.75^/d) along 24 hours of fasting of the broiler breeders previously submitted to different feed regimens

Treatment	Parameters	Estimate	Standard error	P	MSE
Restricted	U (kJ/d^2^)^1)^	7.00	1.544	<0.01	439
	R (h)^2)^	11.95	1.124		
	L (kJ/kg^0.75^/d)^3)^	300	2.619		
*Ad libitum*	U (kJ/d^2^)	10.05	1.417	<0.01	533
	R (h)	11.63	0.726		
	L (kJ/kg^0.75^/d)	364	2.657		

P, probability; MSE, mean square of the error.

Broken line model for the heat production in funtion of the time along the fasting period: HP = U×(t<R)×(R−t)+L

1) Rate of heat production decreasing after the feed was withdrawn.

2) Time of broken point.

3) Plateau value of FHP.
